# The use of electronic health records for recruitment in clinical trials: a mixed methods analysis of the Harmony Outcomes Electronic Health Record Ancillary Study

**DOI:** 10.1186/s13063-021-05397-0

**Published:** 2021-07-19

**Authors:** Emily C. O’Brien, Sudha R. Raman, Alicia Ellis, Bradley G. Hammill, Lisa G. Berdan, Tyrus Rorick, Salim Janmohamed, Zachary Lampron, Adrian F. Hernandez, Lesley H. Curtis

**Affiliations:** 1 Duke Clinical Research Institute, Durham, NC USA; 2 Department of Population Health Sciences, Duke University School of Medicine, 215 Morris Street, Suite 210, Durham, NC 27701 USA; 3 UCB, Durham, NC USA; 4 GlaxoSmithKline, Durham, NC USA; 5 Department of Medicine, Duke University School of Medicine, Durham, NC USA

**Keywords:** Electronic health records, Clinical trials, Screening, Recruitment

## Abstract

**Background:**

The electronic health record (EHR) contains a wealth of clinical data that may be used to streamline the identification of potential clinical trial participants. However, there is little empirical information on site-level facilitators of and barriers to optimal use of EHR systems with respect to trial recruitment.

**Methods:**

We conducted qualitative focus groups and quantitative surveys as part of the EHR Ancillary Study, which is being conducted alongside the multicenter, global, Harmony Outcomes Trial comparing albiglutide to standard care for the prevention of cardiovascular events in type 2 diabetes. Subject matter experts used findings from focus groups to draft a 20-question survey examining the use of the EHR for participant identification, common site recruitment strategies, and variation in perceived barriers to optimal use of the EHR. The final survey was fielded with 446 site investigators actively enrolling participants in the main trial.

**Results:**

Nearly two-thirds of respondents were study coordinators (63.2%), 23.1% were principal investigators, and 13.7% held other research roles. Approximately half of the respondents reported using the EHR to find potential trial participants. Of these, 79.4% reported using EHR searches in conjunction with other recruitment methods, including reviewing of upcoming clinic schedules (75.3%) and contacting past trial participants (71.2%). Important barriers to optimal use of the EHR included the lack of availability of certain research-focused EHR modules and limitations on the ability to contact patients cared for by other providers. Of survey respondents who did not use the EHR to find potential participants, one-quarter reported that the EHR was not accessible in their country; this finding varied from 2.6% of respondents in North America to 50% of respondents in the Asia Pacific.

**Conclusions:**

While EHR screening was commonly used for recruitment in a cardiovascular outcomes trial, important technical, governance, and regulatory barriers persist. Multifaceted, scalable, and customizable strategies are needed to support the optimal use of the EHR for trial participant identification.

**Trial registration:**

ClinicalTrials.gov NCT02465515. Registered on 8 June 2015

**Supplementary Information:**

The online version contains supplementary material available at 10.1186/s13063-021-05397-0.

## Background

The electronic health record (EHR) is a rich source of data that holds promise for streamlining clinical trial conduct. As the primary source of clinical information about patients seeking care in a given system, EHRs contain a wealth of data that may be used to identify potentially eligible patients for clinical trial enrollment. An increasing body of evidence demonstrates potential opportunities with EHR-based data acquisition [1], yet little information is available on site-level barriers to optimal use of EHR systems in contemporary trials, particularly with respect to screening and enrollment. Since each EHR implementation is distinct [2], successful conversions of EHR-screened populations into clinical trial participants presents numerous challenges, including heterogeneous data structures, selection bias introduced by searches focused on care-seeking populations, and limited translatability of inclusion and exclusion criteria [3]. While these challenges are known, few studies have empirically assessed the actual and perceived utility of the EHR for facilitating clinical trial enrollment or barriers to optimal use of the EHR for trial participant identification.

The Harmony Outcomes Ancillary Study is a collaboration between GlaxoSmithKline (GSK) and the Duke Clinical Research Institute (DCRI) to address these knowledge gaps in the setting of a large cardiovascular outcomes clinical trial (Harmony Outcomes) [4]. The primary goals of our analysis were 3-fold: (1) to describe existing site-level processes for using the EHR to identify and screen potential participants for an ongoing clinical trial, (2) to ascertain information on successful recruitment strategies and key barriers to using the EHR for trial recruitment from the perspective of site coordinators, and (3) to evaluate variation in perceived barriers to the use of the EHR by empirical recruitment patterns in the Harmony Outcomes Trial.

## Methods

### Harmony EHR Ancillary Study

The EHR Ancillary Study is being conducted alongside the Harmony Outcomes trial, a multicenter, global, randomized, double-blind, placebo-controlled phase 4 study to evaluate the effect of albiglutide when added to standard therapies on major adverse cardiovascular events in patients with type 2 diabetes mellitus (ClinicalTrials.gov ID: NCT02465515). The Harmony Outcomes trial is complete, and the results have been published [5]. The EHR Ancillary Study has 3 main objectives: (1) to understand how EHR data are used to facilitate trial recruitment and the barriers to that use, (2) to evaluate the fitness of EHR data for use in populating the baseline characteristics in the electronic case report form (eCRF) of the trial, and (3) to explore the fitness of EHR data for use in finding clinical endpoints of interest. The present analysis includes the primary results from the qualitative and quantitative aims of the first objective.

### Qualitative focus groups

Qualitative focus groups and 1-on-1 interviews were conducted via telephone from November 2015 to April 2016. The primary objective of the focus groups was to identify the themes of interest that could be developed into questions for the quantitative survey (below). Focus group participants were recruited from sites expressing an interest in a broad email announcement describing the opportunity to participate in EHR-based focus groups. Focus group participants included 17 study coordinators and 1 site principal investigator at sites actively participating in the Harmony Outcomes trial. Focus group participants represented research sites in four countries (the USA, the UK, Canada, and Denmark), with the majority based in multi-physician or hospital-based practices. Group interviews were conducted by a professional moderator using a semi-structured, open-ended topic guide. For participants unable to attend scheduled group discussions, 1-on-1 interviews were conducted using the same topic guide and methods. Interviews lasted for 1 h on average and were digitally recorded and transcribed. Focus group and 1-on-1 interview data were integrated into a combined dataset for qualitative analysis. We used thematic content analysis to identify key barriers to and facilitators of the use of the EHR for clinical trial participant screening. These results were then used to generate questions on the key themes for the quantitative survey.

### Survey development

Survey questions were drafted by a team of epidemiologists, cardiologists, health services, and clinical trial operations managers based on the results from the focus groups and subject matter expertise. The survey was tested with a small group of DCRI study coordinators (*n* = 6) for interpretability and ease of administration, and refinements to questions and response options were made following this initial testing. Likert scales and multiple-choice items were used to capture information on the recruitment methods most commonly used, perceived effectiveness of each method, frequency of and criteria used in EHR searches, barriers to optimal use of the EHR for recruitment, and reasons for not using EHR searches among sites not currently using this method. The final survey consisted of 20 questions and is provided in the Supplementary Materials.

### Survey recruitment

All actively enrolling sites in the HARMONY Outcomes trial with an EHR were invited to complete the survey. Site contacts for all participating trial sites (*n* = 610) were sent a link to the survey from the sponsor’s country-level study managers with instructions to either complete the survey themselves (if they were involved with trial screening) or to identify an appropriate contact familiar with HARMONY Outcomes screening processes who could complete the survey. An overview of the survey and its purpose was also included on an executive committee call, with a request for committee members to encourage survey completion by participating sites. Sites were asked to complete the survey within a 2-week period and asked to complete only one survey per site. The DCRI and GSK Site Management teams sent two email reminders to site contacts to complete the survey if it was not completed after 2 weeks, and the survey was closed 1 week later. Of 610 enrolling sites, n = 446 (73.1%) had at least one completed survey. Of those who completed the survey, n = 42 had multiple responses. Because survey responses were generally consistent within sites submitting multiple surveys, we retained the first survey completed for analysis.

### Quantitative data analysis

Categorical variables are presented as number (percentage) and group comparisons using the conventional chi-square test. For continuous variables, the sample size and mean ± standard deviation were provided. We limited the present analysis to complete surveys, defined as surveys where respondents reached the end of the survey (hitting the last available “Next/Submit” button). Despite finishing a survey, it was possible for respondents to leave individual questions unanswered. Surveys with unanswered questions were included in the analysis, and the number of responses for each completed question is specified for each result, table, figure, or listing. Given that focus group participants identified a possible association between reasons for non-enrollment and the “yield” of EHR-based searches, we generated *p*-values based on the null hypothesis that the proportion of each specific reason for non-enrollment did not differ across categories of % of participants identified through EHR screening. *P*-values are two-sided, and p < 0.05 was considered to be statistically significant for all analyses. The SAS software (version 9.4, Cary, NC) was used for all analyses. The Duke Institutional Review Board (IRB) provided ethical oversight for the study.

## Results

Qualitative focus groups and one-on-one interviews yielded several key themes that were used to create the final qualitative survey. First, the majority of focus group participants noted that EHR was the primary modality used for screening as well as the highest yield method. Second, focus group participants identified key advantages of EHR-based searches, including the ability to create complex queries, fewer screen failures, and the ability to send messages to non-study doctors to recruit potential trial participants. Third, several key barriers were noted, including restrictions on access to medical records and a lack of research modules designed to support screening. Finally, focus group participants with more experience recruiting participants prior to widespread EHR use noted that electronic searches represented an overall improvement in the screening and recruitment process relative to non-EHR-based methods.

A total of 446 complete surveys were obtained and used in this analysis. Survey respondents were primarily study coordinators (63.2%) followed by site principal investigators (23.1%) and others (13.7%). The highest proportion of respondents reported that they recruited for 2–3 studies at any given time (41.9%), followed by 4–5 studies (27.6%), more than 7 studies (12.8%), 0–1 study (10.8%), and 6–7 studies (7.0%).

Table 1 displays the reported use and perceived yield of potentially eligible patients associated with common recruitment strategies. The 3 strategies that respondents were most likely to report they did not use at all included sending mass mailings informing patients about a trial (61.2%), accessing a community database for people who are interested in research (58.7%), and advertising campaigns online, on local radio stations, or in local newspapers (57.4%). The 3 strategies that respondents were most likely to report using included contacting past clinical trial participants, reviewing upcoming clinic schedules, and asking healthcare providers to help identify patients fitting the trial criteria. Strategies perceived to produce high-yield patients included contacting past trial participants (46.0%), reviewing upcoming clinic schedules (38.8%), and EHR searches (33.6%). Strategies reported to produce low-yield patients included posting flyers in clinics or at local pharmacies and asking healthcare providers to help identify patients.

**Table 1:** Reported use and perceived yield (low, moderate, or high) of potentially eligible patients

Method	Perceived yield (%), ***N*** = 446			
	Do not use this method	Low	Moderate	High
Contacting past clinical trial participants	7.6	12.3	34.1	46.0
Reviewing upcoming clinic visit schedules	10.1	14.3	36.8	38.8
Asking healthcare providers to help identify patients who fit the trial criteria	16.1	32.5	35.7	15.7
EHR searches	26.9	13.0	26.5	33.6
Paper medical record reviews	28.7	16.4	27.6	27.4
Posting flyers in clinics or at local pharmacies	52.2	32.1	13.7	2.0
Advertising campaigns online, on local radio stations, or in local newspapers	57.4	21.1	15.9	5.6
Accessing a community database for people who are interested in research	58.7	19.7	13.2	8.3
Sending mass mailings (electronic or postal) informing patients about the trial	61.2	22.9	12.3	3.6

*EHR*, electronic health record

Nearly half of survey respondents (48.0%) reported using EHR searches to identify potential trial participants. This was consistent among sites in the top 10% of total patients randomized in the Harmony Outcomes trial, 47.7% of whom used EHR searches to identify participants. Of survey respondents using EHR searches to identify participants, the highest proportion used these searches for every trial (45.8%), followed by most trials (33.2%), half of the trials (11.2%), and few trials (9.8%). Nearly four of every five respondents reported using EHR searches in conjunction with other recruitment methods (79.4%). Of these, methods most commonly used in conjunction with EHR searches included reviewing upcoming clinic schedules (75.3%), contacting past trial participants (71.2%), and asking healthcare providers to identify patients who fit trial criteria (65.3%).

Respondents reported that EHR search parameters most commonly contained clinical conditions (88.8%), medications (76.6%), lab parameters (72.4%), and age (71.5%). The largest proportion of respondents reported creating EHR-generated lists of potential participants on a weekly basis (35.0%) compared with less frequent list generation. This proportion was substantially higher among the top 10% of enrolling sites (61.9%). Most respondents reported that EHR searches resulted in fewer than 100 potential participants (32.7% produced < 25, 28.0% between 25 and 49, and 17.3% between 50 and 99).

Table 2 displays the distribution of perceived barriers to the best possible use of the EHR for clinical trial recruitment. For each potential barrier, fewer than 10% of respondents reported that it was a complete barrier to the best possible use of the EHR. Most respondents reported that institutional limitations on accessing the EHR without patient consent presented only a slight barrier or no barrier at all, with similar patterns for institutional limitations on directly contacting patients without their consent. Barriers most commonly reported to be “significant” or “complete” included lack of availability of certain research-focused EHR modules and limitations on the ability to contact patients cared for by other providers.

**Table 2:** Perceived barriers to the best possible use of the EHR for clinical trial recruitment (*N* = 214) (%)

Reason	Not a barrier at all	Slight barrier	Moderate barrier	Significant barrier	Complete barrier
Limitations on ability to contact patients cared for by other providers	30.4	22.9	23.8	15.4	7.5
Certain research-focused EHR modules (i.e., “research and reporting” modules) are not available on our system	32.7	20.1	22.4	15.9	8.9
Not enough IT support to search the EHR	37.9	21.0	25.7	12.6	2.8
Institutional limitations on ability to directly contact patients without their consent	43.0	17.3	21.0	10.7	7.9
Institutional limitations on accessing EHR without patient consent	48.6	14.5	18.7	13.6	4.7

*EHR*, electronic health record; *IT*, information technology

Table 3 displays the most commonly reported reasons that EHR-identified patients did not end up enrolling in the trial. These reasons are displayed overall and are stratified by the proportion of all trial participants that the site reported identifying using EHR searches (0–10%, 11–30%, or > 30%). Reasons commonly identified as contributing a “moderate,” “significant,” or “very significant” amount to lack of enrollment of EHR-identified patients included patient refusal and inability to query some trial criteria. The highest proportion of respondents reported that they enrolled 0–10% of participants using EHR searches (39.3%), followed by 11–30% (33.2%), and > 30% (27.6%). Reasons for non-enrollment were relatively consistent across groupings of percent enrolled, with a slightly lower proportion of respondents in the > 30% enrolled category reporting that non-enrollment was due to inability to query certain trial criteria. However, this difference was not significant at α = 0.05.

**Table 3:** Reported reasons for non-enrollment of EHR-identified patients and by % of total trial participants identified through EHR screening

Reason for non-enrollment	% of total participants identified through EHR screening				
	Overall (***N*** = 214)*	0–10% (***n*** = 84)	11–30% (***n*** = 71)	> 30% (***n*** = 59)	***P***-value
Patient refusal	69.6	71.4	71.8	64.4	0.59
Some trial criteria could not be queried or were not included in the EHR search, and the patient is ineligible	65.0	67.9	70.4	54.2	0.12
Provider deems patient inappropriate for trial	45.8	42.9	47.9	47.5	0.79
Patient is cared for by another provider	36.0	44.0	26.8	35.6	0.08
Patient does not have an upcoming clinic visit	30.8	28.6	32.4	32.2	0.85

*EHR*, electronic health record

*Counts are the number of respondents who selected “a moderate amount,” “a significant amount,” or “a very significant amount” for each reason why patients enroll within each percent enrolled category. The “other” category was taken from question 18. The results are presented for those that answer “yes” to question 18a

**Fig. 1:**
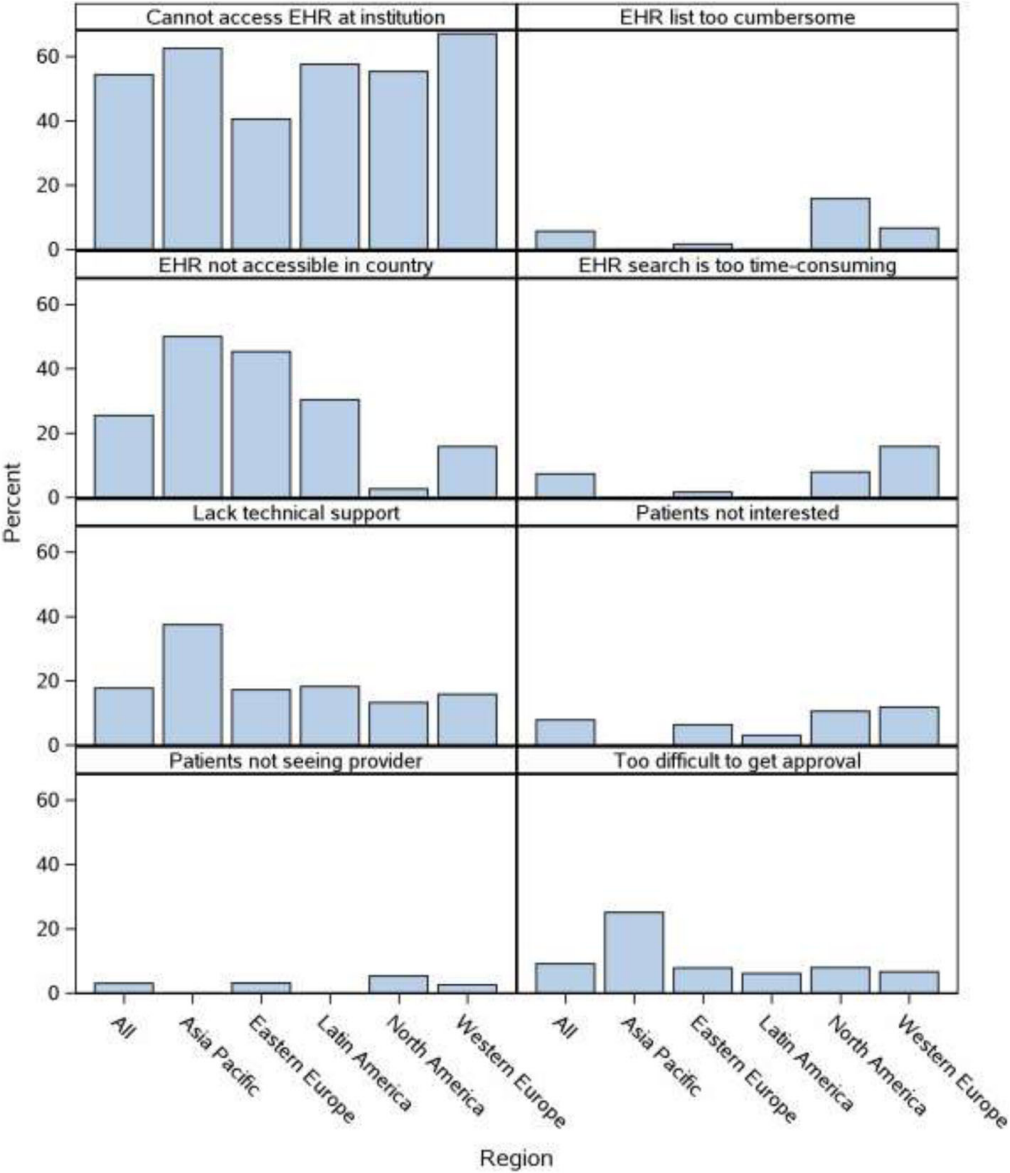
Reasons for not using EHR to search for potential trial participants overall and by region: cannot access EHR at institution = “I cannot access EHR at my institution”; EHR not accessible in the country = “EHR is generally not accessible in my country”; lack technical support = “There is no sufficient technical support to obtain a list of potential participants”; EHR search is too time-consuming = “Conducting an EHR search is too time-consuming”; EHR list too cumbersome = “The list generated from an EHR search is too cumbersome/overwhelming”; patients not seeing the provider = “Many patients identified through EHR search are not seeing a provider I work with”; patients not interested = “Many patients identified through EHR search are not interested in participating”; and too difficult to get approval = “It is too difficult to get approval to do this sort of mass screening.” EHR, electronic health record

Specific reasons for not using the EHR to search for potential trial participants are displayed overall and by geographic region in Fig. 1. The most commonly reported reason was the inability to access the EHR at the respondent’s institution. More than 25% reported that EHRs were generally not accessible in their countries, while 17.7% reported that they did not have the technical support needed to search the EHR. Inability to access the EHR at the institution level was most commonly cited for sites in Western Europe and the Asia Pacific. More broad limitations on accessing the EHR at the country level were reported by respondents at sites in the Asia Pacific and Eastern Europe. The lack of technical support was commonly reported as a contributing reason among respondents from the Asia Pacific.

## Discussion

We conducted a survey of site investigators (coordinators and PIs) actively enrolling in a global cardiovascular trial to address knowledge gaps regarding the use of the EHR for clinical trial recruitment. Our main findings were as follows: (1) nearly half of the survey respondents reported using EHR searches to identify potential trial participants, with the majority reporting that they used EHR searches for most or every trial; (2) most respondents reported using EHR searches in conjunction with other methods, such as reviewing clinic schedules and contacting past trial participants; (3) the most important identified barriers to optimal use of the EHR included the lack of access to research-focused EHR modules and inability to contact patients cared for by other providers; (4) the most important reasons for non-enrollment of EHR-identified patients included patient refusal and inability to query certain trial criteria; and (5) inability to access the institution’s EHR was the most commonly cited reason for not conducting EHR searches, with variation in access to the EHR by geographic region.

Slow or inefficient recruitment remains among the most important challenges to successful clinical trial conduct, with 86% of trials falling behind planned recruitment schedules and 40% failing to meet recruitment goals [6, 7]. Inadequate recruitment can negatively impact study validity by compromising the generalizability of findings and reducing statistical power [6]. By providing access to detailed clinical information on patients receiving care in a given health system, the EHR represents a powerful resource for streamlining the recruitment process through the rapid identification of potential participants [8]. Electronic search methods are less time-consuming and expensive than manual chart review [9] and may reduce screening-related workload by up to 90% [10]. Nonetheless, recent work suggests that even among individuals who commonly use electronic databases for recruitment, “finding eligible patients” remains the most significant barrier to recruitment [11]. The present study addresses an evidence gap regarding the perceived utility of and barriers to EHR-based recruitment from the perspective of active trial investigators.

We found that about 50% of respondents did not use EHR searches to identify potential trial participants and that EHR searches were the fourth most commonly reported recruitment method, reported less frequently than “contacting past clinical trial participants,” “reviewing upcoming clinic schedules,” and “asking providers to help identify eligible participants.” This contrasts with the findings from a survey of 90 stakeholders in the Clinical Trials Transformation Initiative (CTTI) Recruitment Project, who rated “identifying patients using medical records” and “identifying patients using hospital-based registries or other databases” as the most commonly used and most effective recruitment methods [11]. While reported less frequently than other methods, nearly 80% of respondents using EHR searches used them for most or every trial. EHR searches were also perceived to yield a high number of eligible patients compared with most other methods, with the exception of contacting past participants and reviewing upcoming schedules. These results are consistent with the findings from an intervention comparing an electronic search strategy to traditional recruitment in the Lifestyle Intervention for the Treatment of Diabetes (LIFT) trial, which reported higher enrollment yield for participants recruited through referrals than among EHR-screened patients [12]. The majority of respondents in our study used the EHR in conjunction with other recruitment methods, most commonly reviewing upcoming clinic schedules and asking healthcare providers to identify patients who fit the trial criteria. This approach aligns with increasing evidence supporting strategies that integrate EHR searches with other methods, such as provider review of medical charts or upcoming clinic schedules. For example, in a single-center intervention integrating an EHR trial-screening workflow into a health information system, users reported increased recruitment rates in 3 of 7 studies examined, in addition to 10 min of saved time per patient [13]. Other interventions have demonstrated recruitment improvements with automatic provider alerts, which cross-reference clinic schedules with eligibility criteria from the patient’s electronic record. Nevertheless, provider-identified barriers to the use of automated alerts persist, including lack of time and limited knowledge of the patient’s eligibility [14]. Future work identifying scalable approaches to automated searching and notifications of patient eligibility is needed.

While many EHR systems support querying of basic patient characteristics such as demographics and primary clinical conditions, querying of complex trial inclusion/exclusion criteria may be more challenging. One study found that 40% of eligibility concepts commonly used in Alzheimer’s clinical trials were not defined in the EHR [15]. In the Action to Control Cardiovascular Risk in Diabetes (ACCORD) trial, an electronic screening strategy performed well in comparison with investigator review with respect to sensitivity (100%), but had a low positive predictive value (13%), suggesting that the algorithm used included many ultimately ineligible patients [16]. In our study, 39.3% of survey respondents indicated that they enrolled fewer than 10% of EHR-identified participants, and that “inability to query some trial criteria” was among the most important identified reasons for non-enrollment of EHR-identified patients. These results underscore the need for standardization of additional, more specific clinical information to support algorithms incorporating a greater proportion of eligibility criteria.

Our study adds to the literature on geographic variation in recruitment methods and access to the EHR for research purposes. Of survey respondents in our study who did not use the EHR to find potential participants, approximately 25% reported that the EHR was not accessible for research purposes in their country; this finding varied from 2.6% of respondents in North America to 50% of respondents in the Asia Pacific. While we examined variation in barriers across broad geographic regions, prior work suggests that attitudes about the acceptability of EHR-based screening vary even within relatively limited geographic regions. In a survey of participants in the European Electronic Health Records Systems for Clinical Research (EHR4CR) initiative, 70% of respondents reported that using EHR data for recruitment would be permissible and 67% believed it would enhance the conduct of trials, but 50% said it would create ethics or governance concerns in their country and may require prior regulatory approval [17]. Consistent with prior work [18], our study highlights the existence of institution-level restrictions that may represent important barriers to EHR-based recruitment. A better understanding of barriers at the national, local, and institutional levels may help support the consistent implementation of electronic screening strategies in clinical trials.

Several limitations to our study are worth noting. First, we conducted qualitative focus groups with volunteers from a set of sites who responded to an email invitation and indicated their interest in participating in EHR-related focus groups. These participants represented sites that were mostly from the USA, so qualitative findings may not fully represent the views of other sites around the globe. Second, we surveyed site coordinators and principal investigators actively recruiting in a large, global, cardiovascular, outcomes trial for type 2 diabetes; these results may not be generalizable to research in other disease areas. Third, each survey was completed by a single investigator at each site, whose views may not represent the perspectives of all active investigators at that site. Additionally, while nearly three-fourths of actively enrolling HARMONY Outcomes sites completed our survey [n = 446/610 (73.1%)], these sites may not fully represent the experiences of non-responding sites. Finally, several survey response options relied on estimates from investigators (e.g., % of EHR-identified participants enrolled). These estimates should be regarded as exploratory, and future work quantifying the yield of EHR-based searching strategies is needed.

## Conclusions

EHR screening was commonly used to identify potentially eligible participants in a cardiovascular outcomes trial; however, optimal use of the EHR is challenged by technical, governance, and regulatory barriers. Future research examining scalable, multifaceted, electronic, recruitment strategies may support streamlined implementation and reduce EHR screening challenges for clinical trials.

## Supplementary Information

**Additional file 1: Supplementary material.** 20 questions of the final survey.

## Data Availability

The data that support the findings of this study are available from GlaxoSmithKline, but restrictions apply to the availability of these data, which were used under license for the current study, and so are not publicly available. Data are however available from the authors upon reasonable request and with permission from GlaxoSmithKline.

## Abbreviations

ACCORD: Action to Control Cardiovascular Risk in Diabetes
CTTI: Clinical Trials Transformation Initiative
DCRI: Duke Clinical Research Institute
EHR: Electronic health record
EHR4CR: European Electronic Health Records Systems for Clinical Research
GSK: GlaxoSmithKline
LIFT: Lifestyle Intervention for the Treatment of Diabetes
