# Supplementary material for: The use of electronic health records for recruitment in clinical trials: a mixed methods analysis of the Harmony Outcomes Electronic Health Record Ancillary Study
Source: Trials. 2021 Jul 19;22:465. doi: 10.1186/s13063-021-05397-0 (PMC8287813; doi:10.1186/s13063-021-05397-0)
Supplement: Supplementary file 1 — Additional file 1: Supplementary material. 20 questions of the final survey. [file 13063_2021_5397_MOESM1_ESM.docx]

**SUPPLEMENTAL MATERIAL**

GSK HARMONY SURVEY - OBJECTIVE 1

Thank you for your support of the Harmony Outcomes Study.  The entire study team is appreciative of your contributions and efforts toward enrolling patients in the study.

As you are aware, the Harmony Outcomes study includes a unique component – an Ancillary Study to evaluate usability of electronic health records (EHR) data in high-quality clinical research.  GSK and DCRI are conducting this exploratory study alongside the main clinical trial to further our understanding of how EHR data can be organized into a query-able format to facilitate a more efficient, reliable, and cost-effective research process.

Further to your excellent contributions to the study to date, we would appreciate it if you would complete a brief survey and share how you use the EHR for clinical research, including what works best as well as the biggest challenges.  This brief survey should take no more than 10 minutes of your time and will be focused on your experiences with trial screening and recruitment using EHRs.  This data will help inform future strategies that aim to improve the use of EHRs for the conduct of clinical trials.

1.  What is your HARMONY Outcomes Trial Site Number?

What is your site's name?

2.  What is your role in the HARMONY Outcomes Trial?

- Site PI
- Study Coordinator
- Other (specify): ____________________

3.  For how many studies are you usually recruiting at any given time?

- 0-1
- 2-3
- 4-5
- 6-7
- More than 7

4.  In your experience, do the following methods yield a low, moderate, or high number of potentially eligible patients?

|  | Do not use this method | Low | Moderate | High |
| --- | --- | --- | --- | --- |
| a. Posting flyers in clinics or at local pharmacies |  |  |  |  |
| b. Reviewing upcoming clinic visit schedules |  |  |  |  |
| c. Sending mass mailings (electronic or postal) informing patients about the trial |  |  |  |  |
| d. Electronic health records (EHR) searches |  |  |  |  |
| e. Contacting past clinical trial participants |  |  |  |  |
| f. Paper medical record reviews |  |  |  |  |
| g. Asking healthcare providers to help identify patients who fit the trial criteria |  |  |  |  |
| h. Advertising campaigns online, on local radio stations or in local newspapers |  |  |  |  |
| I. Accessing a community database for people who are interested in research |  |  |  |  |

5.  Do you use any trial recruitment methods other than those listed above?

- Yes
- No

Please describe the most common additional trial recruitment method you use and indicate whether it yields a low, moderate, or high number of potentially eligible patients.

|  | Low | Medium | High |
| --- | --- | --- | --- |
| Other (please describe): |  |  |  |

6.  Do you use the same methods when recruiting from inpatient settings and outpatient settings?

- Yes
- No

For the following questions, "EHR search" refers to a process where the user inputs criteria (e.g., age, diagnosis, lab values), to generate a list of potential study participants.

7.  Do you currently use EHR searches to identify potential trial participants?

- Yes
- No

8.  For how many of your trials do you typically use an EHR search to identify potential participants?

- Every trial
- Most trials
- About half of trials
- A few trials
- No trials

9.  When you use an EHR search to identify potential trial participants, do you typically use it alone or in conjunction with other methods?

- Alone
- In conjunction with other methods

If "in conjunction with other methods", which other methods?

- Posting flyers in clinics or local pharmacies
- Reviewing upcoming clinic visit schedules
- Sending mass mailings (electronic or postal) informing patients about the trial
- Contacting past clinical trial participants
- Paper medical record reviews
- Asking healthcare providers to identify their patients who fit the trial criteria
- Advertising online, on local radio stations or in local newspapers
- Accessing a database of people who are interested in research
- Other (please describe): ____________________

10.  What criteria/parameters do you typically use in the EHR search?  (check all that apply)

- Age
- Gender
- Medications
- Upcoming clinic visit dates
- Previous clinic visit dates
- Whether the patient is part of the Principal Investigator's practice
- Lab parameters
- Clinical conditions
- Other (specify): ____________________

11.  How frequently during the trial recruitment period do you create/obtain a list of potential participants from an EHR search?

- Never
- Daily
- Weekly
- Monthly
- Less than monthly

12.  How many potential trial participants are IDENTIFIED from a typical EHR search?

- Fewer than 25
- 25-49
- 50-99
- 100-199
- 200-299
- 300+

13.  Of the total number of potential trial participants identified in a typical EHR search, what % on average ultimately ENROLL in the clinical trial?

- 0-5%
- 6-10%
- 11-20%
- 21-30%
- >30%

14.  In your opinion, the list of potential trial participants that comes from an EHR search for recruitment is generally:

- Too narrow
- About right
- Too broad

15.  In your opinion, please indicate whether the following represent barriers to the best possible use of EHR for clinical trial recruitment:

|  | Not a barrier at all | Slight barrier | Moderate barrier | Significant barrier | Complete barrier |
| --- | --- | --- | --- | --- | --- |
| a. Not enough IT support to search the EHR |  |  |  |  |  |
| b. Institutional limitations on accessing EHR without patient consent |  |  |  |  |  |
| c. Certain research-focused EHR modules (i.e., "research and reporting" modules) are not available on our system |  |  |  |  |  |
| d. Institutional limitations on ability to directly contact patients without their consent |  |  |  |  |  |
| e. Limitations on ability to contact patients cared for by other providers |  |  |  |  |  |

16.  Have you encountered any barriers to the best possible use of EHR other than those listed above?

- Yes
- No

Please describe and rate the most important additional barrier below.

|  | Slight barrier | Moderate barrier | Significant barrier | Complete barrier |
| --- | --- | --- | --- | --- |
| Other (please describe): |  |  |  |  |

17.  We are interested in the process by which the list of potential patients from an EHR search is narrowed to the set of patients who actually enroll in the trial, sometimes referred to as the "enrollment funnel".


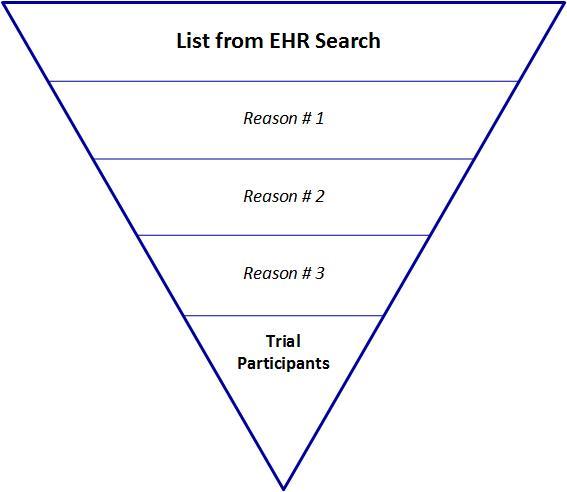


In your experience, how much do the following reasons contribute to EHR-identified patients NOT enrolling in the clinical trial?

|  | Not at all | A little | A moderate amount | A significant amount | A very significant amount |
| --- | --- | --- | --- | --- | --- |
| Patient is cared for by another provider |  |  |  |  |  |
| Some trial criteria could not be queried or were not included in the EHR search and patient is ineligible |  |  |  |  |  |
| Patient does not have an upcoming clinic visit |  |  |  |  |  |
| Provider deems patient inappropriate for trial |  |  |  |  |  |
| Patient Refusal |  |  |  |  |  |

b. Have you encountered reasons that contribute to EHR-identified patients NOT enrolling in the clinical trial other than those listed above?

- Yes
- No

c. Please describe and rate the most important additional reason that contributes to EHR-identified patients NOT enrolling in the clinical trial.

|  | A little | A moderate amount | A significant amount | A very significant amount |
| --- | --- | --- | --- | --- |
| Other (please describe): |  |  |  |  |

For study coordinators who DO NOT currently use EHR to search for potential trial participants:

18.  Please indicate the reasons for not using EHR to search for potential trial participants (check all that apply):

- I cannot access EHR at my institution
- EHR is generally not accessible in my country
- There is not sufficient technical support to obtain a list of potential participants
- Conducting an EHR search is too time-consuming
- The list generated from an EHR search is too cumbersome/overwhelming
- Many patients identified through EHR search are not seeing a provider I work with
- Many patients identified through EHR search are not interested in participating
- It is too difficult to get approval to do this sort of mass screening
- Other (please describe): ____________________

19.  Do you anticipate using EHR to search for potential trial participants in the future?

- Yes
- No
- Not sure
